# Nanoparticles Targeting Macrophages as Potential Clinical Therapeutic Agents Against Cancer and Inflammation

**DOI:** 10.3389/fimmu.2019.01998

**Published:** 2019-08-21

**Authors:** Guorong Hu, Mengfei Guo, Juanjuan Xu, Feng Wu, Jinshuo Fan, Qi Huang, Guanghai Yang, Zhilei Lv, Xuan Wang, Yang Jin

**Affiliations:** ^1^Key Laboratory of Respiratory Diseases of the Ministry of Health, Department of Respiratory and Critical Care Medicine, Tongji Medical College, Union Hospital, Huazhong University of Science and Technology, Wuhan, China; ^2^Department of Thoracic Surgery, Tongji Medical College, Union Hospital, Huazhong University of Science and Technology, Wuhan, China

**Keywords:** macrophages, nanoparticles, drug delivery, inflammation, tumor

## Abstract

With the development of nanotechnology, significant progress has been made in the design, and manufacture of nanoparticles (NPs) for use in clinical treatments. Recent increases in our understanding of the central role of macrophages in the context of inflammation and cancer have reinvigorated interest in macrophages as drug targets. Macrophages play an integral role in maintaining the steady state of the immune system and are involved in cancer and inflammation processes. Thus, NPs tailored to accurately target macrophages have the potential to transform disease treatment. Herein, we first present a brief background information of NPs as drug carriers, including but not limited to the types of nanomaterials, their biological properties and their advantages in clinical application. Then, macrophage effector mechanisms and recent NPs-based strategies aimed at targeting macrophages by eliminating or re-educating macrophages in inflammation and cancer are summarized. Additionally, the development of nanocarriers targeting macrophages for disease diagnosis is also discussed. Finally, the significance of macrophage-targeting nanomedicine is highlighted, with the goal of facilitating future clinical translation.

## Introduction

Macrophages are components of innate immunity and are divided into two types according to their phenotype and function, classically activated macrophages (M1 macrophages), and alternatively activated macrophages (M2 macrophages), that secrete multiple cytokines and express respective surface markers after polarization (1). M1 macrophages express the markers CD86, nitric oxide synthase (NOS) 2, tumor necrosis factor (TNF)-α, and IL-1β. In addition, M2 macrophages highly express the scavenger receptors CD163 and CD200R and release IL-10 (2). M1 macrophages mainly mediate pro-inflammatory processes that protect against the invasion of foreign bodies and play important roles in antitumor immunity in the tumor microenvironment (TME). Furthermore, M2 macrophages possess anti-inflammatory activity in inflammatory diseases but have also been shown to develop protumor characteristics and promote tumor growth and metastasis (3, 4). Macrophages existing in the TME are also called tumor-associated macrophages (TAMs), which suppress antitumor immunity (5). Mononuclear cells in the blood migrate to the tumor site and transform into TAMs, whose phenotype is similar to that of M2 macrophages (2, 6, 7). Therefore, novel therapeutics targeting M1 macrophages in inflammation and M2 macrophages in cancer that efficiently cure these diseases are desirable strategies that should be developed.

Nanomedicine, which has been used in the generation of therapeutic agents, is anticipated to help researchers solve more clinical problems. Drug delivery systems based on nanoparticles (NPs) have been widely used after several decades of technological developments (8). NPs constitute a large family of materials; synthetic NPs with various structures have been produced using a wide range of materials, including liposomes (9, 10), chitosan (11), poly(lactic-coglycolic acid) (PLGA) (12, 13), dextran (14), silica (15), and metals such as iron oxide or gold (16). These materials share several features, including their size range, hydrophilic properties and charge characteristics, which allow them to function as carriers for the delivery of drugs. Moreover, the use of NPs as drug carriers is a novel method to treat certain diseases and has several advantages. First, synthetic nanoscale materials, which are non-human components, are usually avirulent and can easily be formed into capsules and films. Second, NPs can penetrate physiological barriers, such as the blood-brain barrier (17), because of their small diameter and sufficient design, which enable their stability in circulation. Third, NPs and their drug loads can be co-encapsulated stably with high consistency. Fourth, NPs can carry engineered (polymeric NPs) antibodies or aptamers based on the target, which enables them to recognize specific cells. These features all support the use of NPs for drug delivery (8, 12, 18).

In this review, we first discuss the properties of NPs as drug carriers. We then introduce the selection of nanomaterials based on clinical need and the benefits of using nano drugs to treat clinical diseases. Afterwards, we highlight the dual roles of macrophage subtypes in inflammation and cancer. The negative impacts of macrophages are mostly attributed to M1 macrophages that modulate inflammation and M2 macrophages that contribute to cancer. Finally, we illustrate how NPs act as carriers to deliver therapeutic agents to macrophage subtypes in a targeted manner to cure inflammation and cancer.

### NPs as Potential Therapeutic Agents

#### Types and Characteristics of NPs

**Figure 1:**
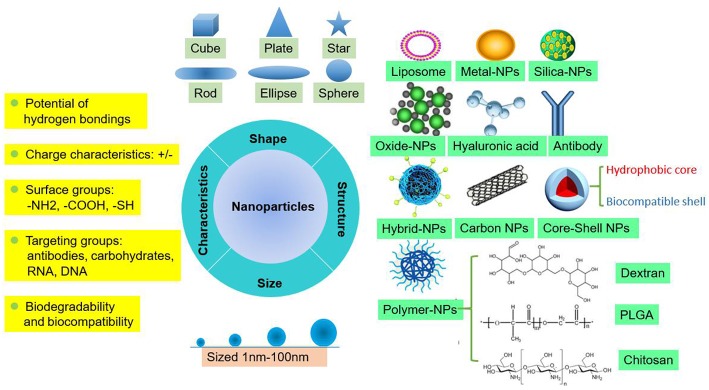
Summary of the fundamental features of NPs as carriers for drug delivery.

NPs are tiny particles (smaller than 100 nanometers) made of materials such as latex, polymers, ceramic particles, metal particles, and carbon particles. NPs are increasingly being used in medical applications due to their physicochemical properties, including their chemical composition, size, shape, structure, morphology, and surface properties (Figure 1). Their surfaces are hydrophilic or hydrophobic and exhibit surface charge and specific ligands, which are reference factors for the selection of nanomaterials used to treat clinical diseases (19). Therefore, NPs play an active role in transporting drugs to the targeted cell and constitute a drug delivery system. Different NPs have been studied by scientists to explore their value for clinical applications.

#### Basis for NPs as Carriers of Therapeutic Agents

When selecting materials for NPs designed to carry therapeutic agents, several limitations of traditional drugs, such as their non-specific distribution, potential toxicity, lack of targeting capability, poor solubility in water and low therapeutic index, need to be taken into account and overcome. NPs must produce metabolites or degradation products that do not harm the human body or are decomposed and eliminated by the body. The degradation products of PLGA-NPs, a relatively mature NP that is currently being explored, are lactic acid and hydroxyacetic acid, which are also byproducts of human metabolic pathways, suggesting that PLGA-NPs can be used in medical applications as a biological material that does not produce toxic side effects. This method has been widely used in the field of biomedicine. Bowerman et al. used PLGA-docetaxel-NPs to treat breast cancer and examined their biodegradability and biocompatibility (20).

Additionally, therapeutic agents loaded into NPs must be designed with optimal sizes, shapes, and surface properties to improve their biodistribution, increase their solubility, enhance their stability, and reduce their immunogenicity (19). For instance, silica-NPs are important nanometer materials, and mesoporous silica NPs (MSNs) are currently being widely investigated. These NPs are non-toxic, tasteless, and do not produce pollution levels similar to those of non-metallic materials, and their high absorptivity and good plasticity make them suitable for disease treatment in the medical field. However, the exploitation of MSNs is currently limited to mouse models for preclinical testing, and the metabolism of silica-NPs is still a problem that hinders successful clinical translation and needs to be researched more deeply (21). In the study by Lu et al., silica was developed into MSNs that were loaded with the antitumor drug oxaliplatin and a specific indoleamine 2,3-dioxygenase inhibitor, which increased the drug concentration at the targeted orthotopic pancreatic ductal adenocarcinoma site in mouse models (22). In the studies by Man et al. and Patel et al., Au-NPs were designed to act as a bidentate ligand to enhance the stability of N-heterocyclic carbene (NHC) ligands (23), and liposomes were exploited for packaging non-water soluble drugs because of their excellent water solubility. These NPs are relatively efficient carriers of short genetic sequences that directly insert genes into cells for assembly (24). A recent phase III trial investigating a lipid NPs (LNPs) siRNA formulation to treat transthyretin (TTR)-induced amyloidosis was successful. Optimized ionizable cationic lipids are a significant factor for the clinical success of LNPs-siRNA. LNPs are the most advanced delivery vehicle, and the Food and Drug Administration (FDA) approved LNPs for the treatment of the hereditary condition TTR-mediated amyloidosis in 2018. Currently, numerous ionizable lipids are available for packaging nucleic acids, which are under clinical investigation for the treatment of various diseases, including cancer, and viral infections (25).

Moreover, designing appropriate NPs for different environments is crucial. For instance, altered pH dynamics, leaky vasculature, and hypoxia are major features in the TME. Nano drugs for acidic environments should be developed to suit different pathophysiological conditions, such as the spatial variations in the TME pH. Chitosan molecules contain free amino acids, can easily form salts in acidic solutions and are cationic, making them useful as drug carriers for antitumor drugs in an acidic TME and for drug release after degradation (11).

#### Clinical Application Prospects of Nano Drugs

Compared to traditional treatments, nano drugs are more beneficial for the treatment of clinical diseases. First, these drugs have an extended plasma half-life in systemic circulation and a prolonged pharmaceutical effect due to their stability in harsh environments, such as the high levels of proteases or other enzymes in the blood stream and the highly acidic environment in the stomach (26). Second, they can alleviate systemic side effects due to their unique mode of drug delivery, which is cell- (such as macrophage) or tissue-specific, and the treatment efficacy can be maximized (27). Finally, nano drugs can control drug release over a manageable period at precise doses (28). Nanomaterials can be combined into groups with different properties to carry different medications for combination therapy, which has the potential to overcome multidrug resistance (19, 29). With the technological development of drug-loaded NPs, the pharmacokinetics and bioavailability analyses of nano drugs indicate that they exhibit characteristics such as vascular permeability, slowed excretion, and mononuclear phagocyte uptake, which is important in the application of NPs-mediated drug delivery systems for disease treatment (30).

The application of these nanomaterials in their respective fields plays a very strong role, and these nanomaterials were designed to emphasize biological compatibility and biodegradable properties. However, we must admit that due to the complex internal environment in the body, most studies performed to date are verified in only animal models, and few are registered in clinical research. Therefore, the application of these materials in clinical medicine needs further research.

### Macrophages Play Different Roles in Inflammation and Cancer

**Figure 2:**
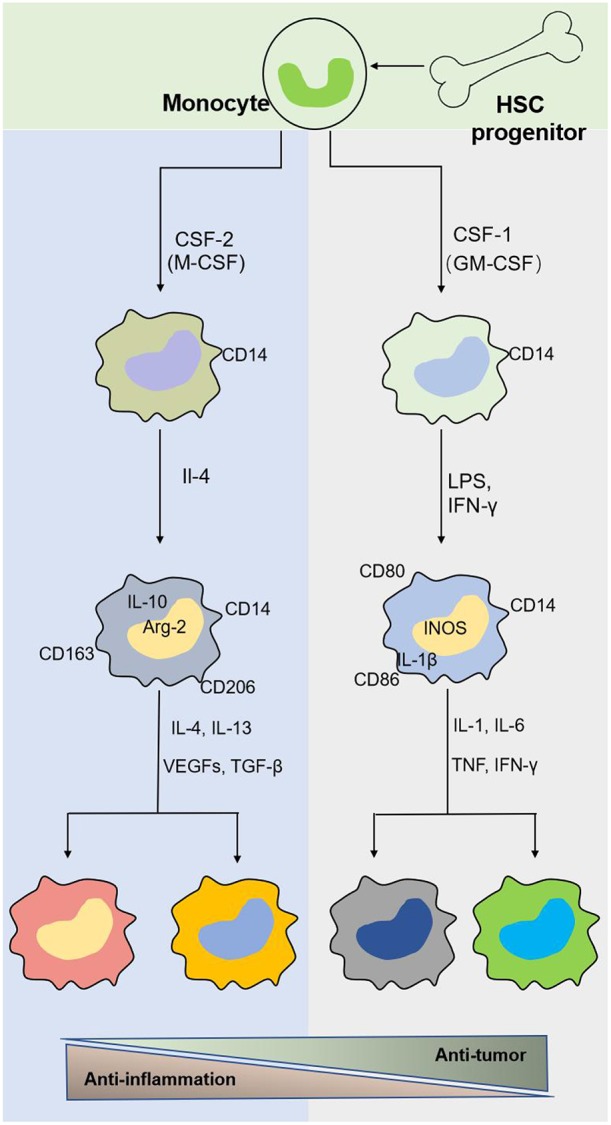
The origin and polarization of macrophages. Tissue macrophages are derived from hematopoietic stem cell (HSC) progenitor cells and exist in blood as monocytes under homeostatic conditions. Monocytes migrate into tissue and differentiate into different macrophages induced by physiologic stimuli, which are associated with a response to inflammatory and cancer conditions.

Macrophages originate from hematopoietic stem cells in bone marrow, present in the blood as monocytes that migrate to tissues and differentiate into mature macrophages of different types under the stimulation of different environments in the body (Figure 2). Different macrophage subtypes have diverse activities in inflammation and cancer. M1 macrophages are key components of inflammation, and they exert their proinflammatory functions by producing high levels of proinflammatory cytokines, reactive oxygen species, inducible nitric oxide synthase (INOS), cyclooxygenase (COX)-2, and reactive nitrogen species. In addition, they also secrete cytokines such as TNF-α, IL-23, IL-1β, and IL-12, which lead to dysfunctional inflammatory responses that develop into conditions characterized by refractory or severe chronic inflammation, such as rheumatoid arthritis (RA) (31), metabolic syndrome-associated disorders (including type 2 diabetes and atherosclerosis), osteoarthritis, asthma, Crohn's disease, and Alzheimer's disease (32, 33). Thus, decreasing proinflammatory cytokine levels at the source may effectively mitigate inflammatory diseases. M1 macrophages play a role in not only proinflammatory immunity but also antitumor immunity, and they play a protective role in tumorigenesis by promoting and amplifying Th1-type responses (34) and secreting a series of cytokines, including TNFs, growth inhibitors, and antiangiogenetic factors. Therefore, M1 macrophages have potential applications as active biocarriers for anticancer drug delivery to tumors.

**Figure 3:**
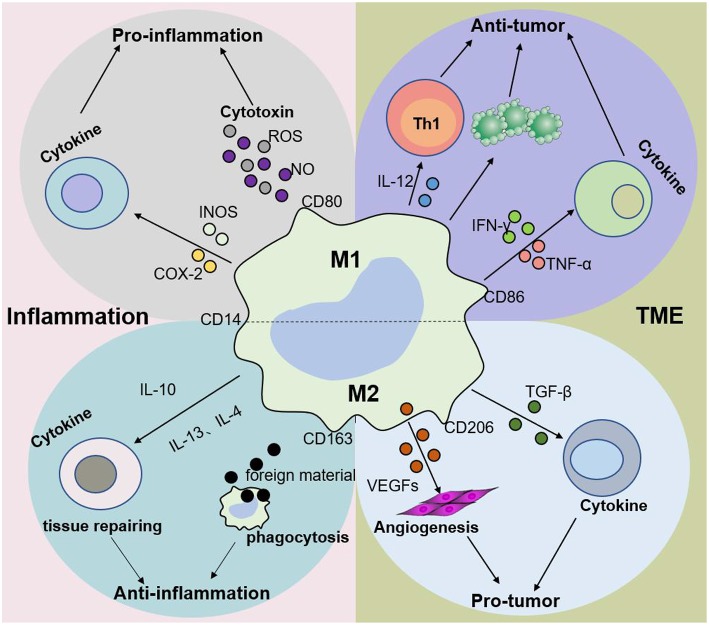
Macrophage subtypes play different roles in inflammation and cancer. In response to various stimuli, such as inflammation, M1 macrophages promote the progression of inflammation, while M2 macrophages mainly play an anti-inflammatory role. Nevertheless, M1 macrophages are mainly involved in antitumor immunity, while M2 macrophages promote tumor growth and invasion in the tumor microenvironment (TME). The dynamic balance between M1 and M2 macrophages jointly determines the evolution of inflammation and tumors.

M2 macrophages participate in antineoplastic immunity by increasing the number of suppressor cells among myeloid-derived suppressor cells (MDSCs), TAMs, and immature monocytes. Macrophages in tumors, also called TAMs, comprise 50% of the TME population and contribute to tumor progression and poor prognosis (35–38). In addition, the percentage of TAMs is inversely proportional to the survival period, i.e., higher TAM numbers are correlated with shorter tumor patient survival (39). Furthermore, M2 macrophages secrete cytokines such as IL-4 or IL-10, IL-13, VEGFs, and TGF-β to participate in the anti-inflammatory response. The effects of macrophages on tumors and inflammation are shown in Figures 2, 3. Based on the dual role of macrophages, nano drugs mainly target negative macrophages in different diseases, delivering relevant drugs to localized areas to change the polarization conditions, thereby leading to a positive conversion between macrophage subtypes.

Although NPs development is progressing quickly, many obstacles, and challenges remain regarding NPs drug delivery systems, which we will now discuss. The first problem that should be addressed is the mononuclear phagocyte system (MPS), which is a family of cells, including bone marrow precursors, blood monocytes, and tissue macrophages (40). The MPS enables the elimination of NPs through phagocytosis. Interestingly, Rodriguez et al. attached “don't eat-me” marker CD47 “self” peptides to the surface of NPs to avoid phagocytosis. As part of the MPS, macrophages become the popular target for its passivity, representing a potential area for elucidating more macrophage-targeting therapeutic methods. After MPS phagocytosis, NPs will enter the blood circulation and must then be cleared (41). Many solutions have been proposed to circumvent this problem, such as the novel multistage delivery vector (MSV) proposed by Blanco et al. aimed at successful delivery in the blood vessel (42, 43). NPs cannot exert their therapeutic effects, involving extravasation, cellular membrane traversal and cellular internalization, until they reached their intended target (44). As the mechanism of NPs drug delivery systems becomes increasingly clear, these obstacles need to be further researched to develop different methods for modifying NPs that can overcome these obstacles.

### Strategies for Targeting M1 Macrophages by NPs to Treat Inflammation

#### Depletion of M1 Macrophages

**Figure 4:**
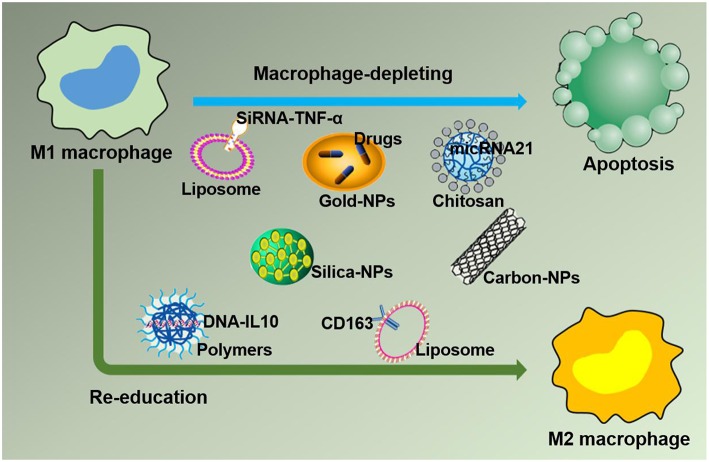
Strategies for nanoparticles packaged with therapeutic agents to target M1 macrophages in inflammation leading to M1 macrophage depletion and re-education.

Based on the negative effect of M1 macrophages on inflammation caused by large numbers of macrophages accumulating at the lesion site, leading to imbalanced inflammation, strategies targeting M1 macrophages in the inflamed tissue with drug-loaded NPs are desirable. In this section, drug-loaded NPs targeting M1 macrophages in the inflammatory environment are discussed. Furthermore, macrophages can quickly and directionally migrate to pathological sites where specific chemokines are being secreted, which allows them to serve as vehicles for the targeted delivery of drugs (45). The strategies of NPs targeting M1 macrophages by delivering specific ligands are illustrated in Figure 4. Two methods currently exist for targeting macrophages: macrophage depletion by NPs-loaded drugs and macrophage re-education by NPs carrying specific cytokines to the microenvironments (46). Thus, in inflammatory disorders, downregulating M1, or repolarizing M1 macrophages to M2 macrophages are two major approaches to relieve inflammation. One example is to use NPs composed of poly(lactic acid)-poly(ethylene glycol) block copolymer (PLA-PEG) to deliver a TNF-α siRNA to M1 macrophages in the model of inflammatory bowel disease (IBD). NPs have been shown to be more powerful and efficient when the Fab' portion of the F4/80 Ab (Fab'-bearing) is grafted onto the NPs surfaces and to attenuate colitis more effectively (47). Interestingly, although LNPs have been extensively studied as delivery systems for nucleic acid therapy, there are also several reports of their application in IBD therapy and targeting macrophages in particular. Recently, Veiga et al. (48) used the Anchored Secondary scFv Enabling Targeting (ASSET) platform to fabricate mRNA-loaded LNPs, which targeted gene expression in Ly6C^+^ inflammatory leukocytes. The authors then determined the potential therapeutic efficacy of IL10 cytokine expression in Ly6C^+^ inflammatory leukocytes in a dextran sodium sulfate (DSS) colitis model. All research results indicated that the novel therapeutic strategy of using mRNA-LNPs to specifically target anti-inflammatory cytokines in inflammation-related cells for the treatment of inflammation-related diseases has a promising development prospect (48). Increasing evidence has shown that macrophages, which produce proinflammatory cytokines such as TNF-α that are conducive to disease progression and/or maintenance, play an important role in the pathogenesis of chronic inflammation. Therefore, TNF-α has become a popular target for IBD therapy. Xiao et al. successfully synthesized a macrophage-targeted bioreducible PPM conjugate and utilized it to fabricate NPs carrying sodium triphosphate (TPP) and siRNA by electrostatic interaction. Macrophages can efficiently assimilate these NPs and release high-level RNAi, which decreases TNF-α expression and exerts anti-inflammatory effects *in vitro* and *ex vivo* (49). Aouadi et al. developed β1,3-D-glucan-encapsulated siRNA particles (GeRPs) as delivery vehicles that silence genes of mouse macrophages. GeRPs can inhibit the production of TNF-α and IL-4 in macrophages by silencing Map4k4, an unknown mediator of cytokine expression in macrophages (50).

In a recent study by Bejerano et al., miRNA-21-loaded NPs delivered miRNA-21 to cardiac M1 macrophages after myocardial infarction and subsequently increased angiogenesis, reduced the number of apoptotic cells, and improved cardiac healing by downregulating the expression of TNF-α and iNOS. This study highlighted a new therapeutic strategy to target M1 macrophages using the NPs-mediated delivery of miRNA-21 to resolve inflammation (51). These methods directly target M1 macrophages with nano drugs to decrease the levels of proinflammatory cytokines and have been proven to be an effective strategy to treat diseases in preclinical model (52). This NPs-based approach should significantly benefit patients suffering from inflammatory diseases when the technology is applied clinically in the future.

#### Re-education of M1 Macrophages

Another novel treatment strategy for chronic inflammatory diseases is repolarizing macrophages from an M1 to an M2 phenotype (Figure 4). RA, an autoimmune disease, manifests as the accumulation of macrophages in the arthritic synovium, which limits drug access and renders RA difficult to treat. Jain et al. attempted to encapsulate the anti-inflammatory (IL-10) cytokine encoding plasmid DNA into non-condensing alginate NPs and then modify the tuftsin peptide to the surface of the nanocarriers to actively target macrophages. This technology enabled nano drugs to easily enter the arthritic synovium to deliver drugs to macrophages and successfully reprogrammed the macrophage phenotype from M1 to M2, which led to the downregulation of proinflammatory cytokine (IL-6, IL-1β, and TNF-α) expression in systemic and joint tissues and eventually prevented the progression of inflammation and joint damage in arthritic rat models (53).

Importantly, NPs expressing targeting ligands themselves or the addition of targeting ligands to the surface enables NPs to specifically target cells through selective binding to the receptors overexpressed on the cell surface. Dextrin may serve as a representative targeting molecule and has been applied as a plasma volume expander in clinical applications due to its high biocompatibility. The development of nanotechnology has increased the potential applications of dextran for the treatment of inflammatory diseases through the synthesis of dextran-NPs that can target macrophages (14). The selective and high efficiency of dextran-NPs at targeting macrophages is due to the expression of dextran-binding C-type lectins and scavenger receptors on their surface, and these NPs are excreted due to metabolic processing (18). Jain et al. already developed novel carriers to transport IL-10 into inflammatory environments to repolarize macrophages from an M1 to an M2 state, which could serve as a novel therapeutic strategy for the treatment of chronic inflammatory diseases (53). Polyethylenimine NPs carrying the gene for CD163 (an M2 macrophage marker) grafted with a mannose ligand can target cells with a monocytic origin, especially M1 macrophages, thereby converting M1 macrophages into M2 macrophages, leading to the release of anti-inflammatory factors to resolve inflammation and the alleviation of inflammatory disease progression (54). Overall, nano drugs are a novel platform for clinical treatments.

### Tactics for Using NPs to Target M2 Macrophages in Cancer Treatment

#### Depletion of TAMs

**Figure 5:**
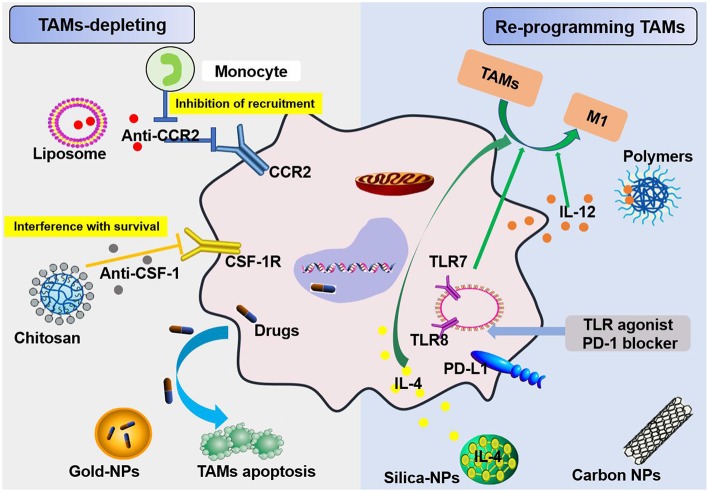
Schemes for nanoparticles loaded with specific agents to target TAM signaling pathways. Nanoparticles target TAMs in the TME via two mechanisms, TAM depletion and TAM reprogramming. The former includes the inhibition of recruitment by blocking the CCL2-CCR2 signaling pathway and survival interference via inhibiting the CSF1-CSF1R signaling pathway. The latter mainly involves cellular re-education to change TAMs into M1 macrophages.

In regard to TAM targeting, strategies can be divided into two categories: depletion of TAMs and reprogramming of TAMs [(55, 56); Figure 5]. Advanced methods to deplete TAMs include the inhibition of colony-stimulating factor 1 (CSF1)–CSF1 receptor (CSF1R) signaling, which contributes to the apoptosis of a large proportion of TAMs (57). Many clinical treatment strategies that target CSF1R have been developed, including the employment of small molecules and antiCSF1R mAbs, and are summarized in Muhammad Ovais' review (58). Another approach to deplete TAMs is blockading the recruitment of circulating inflammatory monocytes to the tumor site. This process is highly dependent on CC-chemokine ligand 2 (CCL2)–CC-chemokine receptor 2 (CCR2) signaling. By inhibiting the CCL2–CCR2 signaling pathway, mononuclear cells remain in the bone marrow, which leads to a decrease in recruitment to tumor primary and metastatic lesions (59–62). Preclinical trials on the CCL2–CCR2 blockade approach are ongoing. Anti-CCL2 antibodies in combination with chemotherapeutics were proven to be effective for advanced metastatic prostate cancer in murine models (63). In addition, a small molecule CCR2 inhibitor (PF-04136309) was applied in a preclinical pancreatic cancer mouse model, which resulted in reduced tumor growth and fewer liver metastases by reducing the recruitment of inflammatory monocytes to the tumors (64).

Recent research suggests that TAMs hinder antitumor therapies by expressing PD-1 and inhibiting acquired immunity, like T cells, as well as by inhibiting innate immunity, similar to macrophages. TAMs that produce PD-1 and exhibit poor phagocytosis are much worse at engulfing tumor cells (65). In addition, the TME downregulates CD47 expression on TAMs, and this molecule was recently shown to be involved in an immune checkpoint. Thus, immunotherapies targeting TAMs are very promising strategies for fighting cancer along with current immunotherapies, including chimeric antigen receptor T cell therapy (CAR-T), T cell receptor therapy (TCR-T), cytotoxic T lymphocyte-associated protein (CTLA)4 antibody therapy, programmed death (PD)-1 therapy, and programmed death-ligand (PD-L)1 antibody therapy (66). Furthermore, paclitaxel is currently one of the best natural anticancer drugs and has been developed as a nano drug and approved for clinical use in the treatment of breast cancer, ovarian cancer, and lung cancer. Paclitaxel-loaded NPs can penetrate tumor microvessels in solid tumors via binding a high-affinity LFA-1 I domain with cross-linked amphiphilic copolymers. The LFA-1 I domain can specifically identify ICAM-1, which is overexpressed in inflammatory and neoplastic conditions in areas such as the vasculature and TAMs. The amphiphilic copolymer acts as a drug carrier due to its hydrophilic corona, which produces stability, and its hydrophobic core, which allows encapsulation of paclitaxel because it is not highly soluble in water. Ultimately, nano drug delivery of paclitaxel targets markers prevalent in carcinomas and an inflamed TME, such as TAMs (67). In 2019, Rybakova et al. (68) used an *in vitro*-transcribed mRNA (IVT-mRNA) system for the *in vivo* delivery of a humanized anti-HER2 (also known as ERBB2) antibody, trastuzumab, and packaged IVT-mRNA into LNPs. This approach not only ensures anticancer activity and efficient *in vivo* delivery but also delays degradation of the mRNA (68). Rafique et al. encapsulated calcitriol into LNPs and developed near-infrared calcitriol PEGylated NPs [PEG-LNP(Cal)] by a microfluidic mixing technique. The results showed that calcitriol can be effectively targeted to macrophages (69). Nanotechnology systems for the targeted delivery of drugs to TAMs are effective methods for mitigating tumor promotion (46). These systems also substantially increase the efficiency of chemotherapy and overcome drug resistance in treating cancer (70).

#### Re-education of TAMs

TAMs are responsible for tumor promotion and malignant tumor development, which is usually associated with poor prognosis. Because TAMs are highly plastic, reprogramming these cells toward a tumoricidal phenotype has been a popular therapeutic strategy.

TAMs promote tumor development through the secretion of immunosuppressive agents. Therefore, TAMs are potential targets for cancer therapy by nano drugs that can reprogram TAMs into antitumorigenic macrophages (71). Reprogramming of TAMs, also called re-education of TAMs, is currently the most attractive strategy for cancer treatment and can reverse the protumor phenotype into the antitumor phenotype (55, 58). This method can slow or stop cancer growth by activating the antitumor functions of M1 macrophages and stimulating the activity of Th1-type cytotoxic T cells and other effector cells (72). In recent years, more researchers have focused on small molecules and NPs formulations for macrophage repolarization, such as Toll-like receptor (TLR) agonists, cytokines, antibodies, and RNAs (73). In addition to the polarization methods mentioned, we summarize the common polarization targets in Table 1. Here, we describe some specific studies that have been performed regarding TAM depolarization. Polymeric NPs have been designed and synthesized with an IL-12 payload to re-educate TAMs, eventually promoting macrophage conversion from the M2 to the M1 phenotype, demonstrating the promise of nanomaterials as a platform for cancer immunotherapy (78). TLR agonists are a potential method to polarize TAMs into M1-like cells (82). NPs loaded with TLR agonists targeting TAMs are currently being tested as antitumoral agents in animal models. In 2018, Rodell et al. established that R848-loaded β-cyclodextrin NPs (CDNP-R848) contributed to efficient drug delivery to TAMs. The authors capitalized on β-cyclodextrin (CD), which enables drug solubilization, and R848, an agonist of TLR7 and TLR8 that can target TAMs and eventually shift TAMs to an M1 phenotype, thereby improving cancer immunotherapy and controlling tumor growth. This method has been utilized in animal models, protecting the animals against tumor rechallenge and improving immunotherapeutic responses when used in combination with the immune checkpoint inhibitor anti-PD-1. This treatment strategy represents a tool to transform TAMs into an M1-like phenotype and subsequently promote tumor regression and increase the efficacy of immune checkpoint blockade (83, 84). Another successful example is the encapsulation of miR-125b into CD44 targeting hyaluronic acid-poly(ethylenimine) (HA-PEI)-based NPs by Parayath et al. After abundant scientific experiments, the authors determined that this type of NPs successfully enables the transfection of TAMs and demonstrated its contribution to TAM repolarization in anticancer immunotherapy (85).

**Table 1:** Summary of the common polarization targets of TAMs.

**Target**	**Signaling pathway**	**Clinical effects**	**References**
TLR	NF-κB/IRF3	TLR agonists are commonly used, such as in the treatment of malignant pleural effusion (MPE) in advanced lung cancer patients	(74, 75)
CSF1	NF-κB/ERK1/2	Anti-CD40/anti-CSF-1R therapy improved antitumor efficacy	(76)
CD40	NF-κB	Anti-CD40 combined with imatinib has clinical potential for the treatment of GIST	(77)
IL12	JAK2/STAT4	IL12 is applied for NPs	(78)
TREM-1	TREM-1/DAP12/Syk	TREM-1 plays a crucial role in regulating IL-22 production by ILC3 through modulating M1 macrophage polarization during DSS-induced acute colitis	(79)
BTK	Blocks BTK	BTK inhibitor is targeted to leukemia in NPs	(80)
COX2	Blocks PI3K/Akt	COX2 inhibitor is approved for leukemia	(81)

More than that, carbohydrates like mannose can also employ to target macrophages. The expression of mannose receptors in the cells of the immune system was preferential which made mannose become a popular ligand targeting macrophages (86). Lots of new methods using mannose as targeting ligand have been proposed in recent months. Wang et al. developed twin-like core shell NPs (TCN) for targeting delivery of sorafenib and TAMs re-polarization agents IMD-0354 to cancer cells and TAMs which could enhance tumor-localized chemoimmunotherapy. Mannose, as a targeting ligand was anchored to IMD-0354 for selective targeting delivery to TAMs (87). Zhao et al. synthesized the albumin NPs modified with dual ligands, a transferrin receptor (TfR)-binding peptide T12 and mannose. They proved that this system efficiently inhibited the glioma cell proliferation and successfully “re-educated” the protumor M2 toward antitumor M1 (88). Most of mannose as ligand targeting macrophages now are applied for macrophage re-polarization. It is helpful and of great potential to be used in tumor-localized chemoimmunotherapy. Additionally, TAMs can activate the nuclear factor (NF)-κB pathway by producing ligands to target protumor genes such as CXCL12 and VEGFC via the CD40 receptor (89). Interestingly, CD40 blockage also leads to the upregulation of IL12, which can repolarize TAMs into M1 macrophages. For instance, NPs loaded with a corresponding inhibitor can block the NF-κB signaling pathway, which can switch TAMs into M1 macrophages that are toxic to tumor cells; this approach has a bright future in cancer treatment (90). Additionally, for successful nanocarrier drug delivery to macrophages, different drugs and their individual physicochemical properties should be considered. The encapsulation strategies, including the covalent stimulus-responsive covalent linking of drugs to the carrier and different release rates, are summarized in Table 2. In all, NPs deliver specific ligands to M2 macrophages to modulate the function in tumors, as summarized in Figure 5.

**Table 2:** Different drug encapsulation strategies.

**Type of drug**	**Specific drug**	**Encapsulation strategy**	**References**
Metal complex formation	Cisplatin	Cisplatin-loaded polymeric micelles (CDDP-PMs) is supposed to improve encapsulation efficiency. Chlorine ligands can be replaced with a polymer s carboxylate group, leading to a stable formulation from which cisplatin can be recovered in physiological conditions. Such a strategy is supposed to improve encapsulation efficiency (vs., for example, that of passive liposomal encapsulation, which does not exceed 18%)	(91–93)
	In and Ga	Empty liposomes are initially loaded with the chelating agent, and immediately prior to administration, the drug is formed *in situ* by incubation with another metal complex with a smaller binding constant, leading to 90% encapsulation of In and Ga in liposomes	(91, 94, 95)
	^225^Ac	^225^Ac is encapsulated by liposomes. After 30 days, ^225^Ac retention as high as 81% of the initially encapsulated radioactivity was achieved	(91, 96)
Electrostatic interactions	Genetic material	Ionizable amino lipids are used as an alternative to cationic lipids to produce liposomes with improved encapsulation ability. These lipids are cationic at the time of preparation in acidic pH but remain neutral at the time of administration (at physiological pH). One example is the use of 1,2-dioleoyl-3-dimethylammonium propane (DODAP), which markedly changes the encapsulation of oligonucleotides from a mere 5% at 0% DODAP to 80% at 30% DODAP in the lipid mixture at pH 4	(91, 97)
	Negatively charged drugs	NPs containing cholesterol and stearylamine encapsulate high payloads of retinoic acid by ionic interactions. The addition of positively charged stearylamine remarkably improves the entrapment from 13% (without stearylamine) to > 90%	(91, 98)
	Positively charged drugs	Using anionic liposomes containing phosphatidylserine can improve the encapsulation of cationic drugs such as cisplatin. Positively charged PLGA NPs contain donepezil hydrochloride (DP) that is used as a positively charged hydrophilic drug model. Then the PLGA NPs are coated with chitosan hydroxy propyltrimonium chloride	(91, 92, 99)
	Zwitterionic and amphiphilic drugs	Polyelectrolytes, such as polyethyleneimine-based polymers, can improve the encapsulation of hydrophobic drugs	(91, 100)
Covalent bonding	PTX	Hu et al. prepared carboxyl-terminated diblock copolymers (mPEG-b-PLA) and triblock copolymers (PLA-bPEG-b-PLA) and attached paclitaxel (PTX) to their molecular ends. By changing the block lengths of PEG and PLA, the paclitaxel content in the conjugates can be widely adjusted up to 8–15% wt	(91, 101)
Hydrogen bonds	5-Fluorouracil	Bhadra and co-workers successfully synthesized dendonized poly(amido amine) polymers to encapsulate drugs such as 5-fluorouracil. By using this strategy, hydrogen bonds played an important role in improving the efficiency of the drug complexation	(91, 102)
Hydrophobic encapsulation	MTX-Oet	Cerqueira et al. produced polyoxyethanyl-α-tocopheryl sebacate (PTS) micelles to deliver a hydrophobic derivative of methotrexate, MTX di-ethylated (MTX-OEt). MTX-OEt was efficiently encapsulated onto the produced PTS micelles which preserved their physicochemical properties. This strategy showed a promising intracellular delivery performance with potentiality for cancer therapy	(103)
	Curcumin	The encapsulation of curcumin (or THC) into thehydroxypropyl (HP)-cyclodextrins (CD) (HP-CDs) significantly increased the drug solubility and enhanced the corneal and retinal epithelial permeability	(104)
	Astaxanthin	Astaxanthin was loaded in poly(lactic-co-glycolic acid) (PLGA) NPs coating with chitosan oligosaccharides (COS). The encapsulation efficiency (>85%) and loading capacity (>15%) of the astaxanthin in the NPs was relatively high	(105)
	(±)-α-Tocopherol (TP)	The (±)-α-Tocopherol (TP) with vitamin E activity was encapsulated into biocompatible core-shell structured NPs which were synthesized by poly(lactic acid) (PLA) and poly(lactide-co-glycolide) (PLGA). This encapsulation strategy obtained NPs with a hydrophobic TP core and a polymer shell with high encapsulation efficiency (EE%) (69%)	(106, 107)

However, when trying to target TAMs in the TME, the infiltration of nano drugs should be considered. To avoid the uptake of infiltrated nano drugs by normal macrophages and promote the retention of nano drugs in tissues, the following methods may be used. First, high interstitial fluid pressure (IFP) is one of the most important features of the TME, as it contributes to NPs accumulation in tumors and restricts their extravasation and penetration (108). Another strategy is utilizing a pH-sensitive design for NPs. Because acidosis is a typical physiological factor of the TME and the tumor pH ranges from 6.5 to 6.8 while the pH in healthy tissues is 7.4, NPs designed to be pH-sensitive can release drugs in tumors and exist stably in normal tissues (19, 109). For instance, polymers, including poly(acryl amide) (PAAm), micelles and liposomes, can release drugs by changing structures in the TME through protonation or deprotonation (110–113). These approaches may prevent NPs from participating in humoral circulation and being ingested by normal tissue macrophages.

Although advanced cancer research has revealed that the TME affects cancer progression and metastasis (71), some specific TME conditions could be used to promote NPs uptake by TAMs in tumors. For example, the enhanced permeability and retention (EPR) effect is a typical characteristic of the TME (114). EPR may be related to the difference between tumor vasculature and normal blood vessels, with leaky capillary gaps of the former being ~100–780 nm and those of the physiological blood vessels being ~5–8 nm (115–117). Many studies have shown that NPs accumulate more in tumors than in other tissues due to the heterogeneity of the tumor vasculature and EPR effects. In addition, the half-life of the drug in the NPs system is prolonged for escapement from renal clearance (118). These two conditions, which encompass the blood circulation stability of NPs and the EPR effect, provide an avenue for drugs to reach and combat tumors. The EPR effect makes NPs extravasate from the tumor vasculature and transport into tumor cells and stroma, which leads to the accumulation of antitumor drugs in the TME at high concentrations and a reduction in systemic side effects. Exploiting this EPR effect and active targeting moieties was shown to be beneficial for the success of NPs-based therapy and for coping with delivery-related drug resistance (119). Therapeutic agents based on NPs targeting TAMs may represent a new approach to curing cancer in the clinic.

We have thus far summarized two methods for targeting macrophages with NPs in these two environments, depletion and re-education of macrophages. Polarizing active M1 macrophages into the immunosuppressive M2 phenotype in an inflammatory environment and repolarizing M2 macrophages into the immunoactive M1 state in the TME appear to be most effective in different circumstances. In fact, beyond the consumption of M1 macrophages in inflammation and TAMs in cancer, their main disadvantage is the loss of their potential immune stimulatory effects as major phagocytes and specialized antigen presenting cells. Therefore, the functional repolarization of macrophages to enhance their beneficial function and limit their detrimental properties is currently the most attractive strategy for disease treatment.

### Diagnostic Purposes of Targeting Macrophages

As stated above, targeting different macrophage subtypes by different NPs can efficiently cure inflammation and cancer in animal models. In addition, macrophage targeting might be used for disease diagnosis, especially for malignant lesions, beyond the delivery of TAM-targeting therapeutic drugs by NPs. However, this approach is linked to the design, development, and application of NPs (120). Based on the type of NPs, the PET, CT, MRI imaging modalities, and their combinations can be used to detect the accumulation of NPs in macrophages (121). For example, MRI-compatible nanomaterials, such as iron oxide NPs, can be rapidly recognized and phagocytosed by macrophages, which leads to a positive MR signal effect on T1-weighted MR images, and the signal strength is proportional to the number of macrophages, thereby providing a non-invasive method to assess the progression and prognosis of diseases and facilitating treatment-related decisions by quantifying TAMs in tumors (122). Interestingly, abnormal patterns of NPs accumulation are helpful for the accurate detection of lymph node metastases in prostate cancer, which is judged by unique MRI features (123, 124). The clinical trial (NCT01770353) of MM-398 (nanoliposomal irinotecan, Nal-IRI) assessing the practicability of ferumoxytol by imaging TAMs to predict patient response is currently ongoing. Furthermore, the binding of various molecular markers of macrophage subtypes to NPs surfaces allows specific recognition by macrophages (125). For instance, NPs-labeled Ly-6C^high^ monocyte biomarkers are more efficient than their Ly-6C^low^ counterparts in mice (126). Therefore, developing non-invasive methods for imaging macrophages holds substantial promise.

## Concluding Remarks

In summary, we report the current status of macrophage-targeting NPs in the context of inflammatory disorders and cancers. Together with the facts that macrophages are naturally involved in and crucial for the pathogenesis of inflammation/cancer and that current nanotechnologies can design and generate NPs that specifically target macrophages, the rich variety of examples discussed above makes us confident that well-engineered NPs targeting macrophages demonstrate a new paradigm in a wide range of therapies for cancer and inflammatory disorders, such as acute lung injury, RA, stroke, atherosclerosis and myocardial infarction.

While NPs targeting macrophages have shown potential in disease treatment, many interesting questions and challenges still need to be addressed. First, as we know, the time course of macrophage infiltration is always dependent on the pathogenesis of the disease and its stages; thus, studies on the dynamics of macrophage activation and trafficking in cancer and inflammatory disorders are needed for optimization of the best time to deliver NPs to better target infiltrated macrophages. Second, recent studies have highlighted the complexity and phenotypic heterogeneity of inflammatory monocyte-derived cells or resident macrophage populations in different tissues and disease contexts (127). As their variability extends far beyond the simplistic classifications of M1 and M2 phenotypes, NPs must be engineered to achieve selective targeting by clearly elucidating and differentiating the specific subtypes. Last but not least, due to the heterogeneity of the inflammatory/tumor microenvironment, NPs–cell interactions are also vital mechanisms that must be elucidated. These NPs–cell interactions, including but not limited to the internalization and processing of nanomaterials by macrophages and other immune cells, are only partially understood and play an important role in both therapeutic and imaging efficacy.

Initial data from macrophage-targeted pharmacological interventions in clinical trials indicate that macrophage-targeting strategies can be successfully translated into novel clinical treatment options (128, 129). However, for the clinical translation of NPs-based macrophage-targeting therapies, further work is required to explore the safety of NPs, patient-specific responses, disease-type specificity, and precise targeting/imaging technology to close the gap between the bench and clinic.

## Author Contributions

GH and MG wrote the draft. YJ reviewed and edited the manuscript before submission. JX, FW, JF, QH, GY, ZL, and XW commented and added extra information.

### Conflict of Interest Statement

The authors declare that the research was conducted in the absence of any commercial or financial relationships that could be construed as a potential conflict of interest.
